# Failure of Real-time Passive Notification about Radiation Exposure to Influence Physician Ordering Behavior

**DOI:** 10.7759/cureus.695

**Published:** 2016-07-15

**Authors:** Lauren A Polen, Jennifer K Rossi, Cameron K Berg, Raymond R Balise, Robert J Herfkens, Paul S Auerbach

**Affiliations:** 1 Emergency Medicine, California Pacific Medical Center, San Francisco; 2 Emergency Medicine, Oregon Health; 3 Emergency Medicine, North Memorial Healthcare; 4 Department of Public Health, Division of Biostatistics, University of Miami Health System; 5 Radiology, Stanford University School of Medicine; 6 Department of Emergency Medicine, Stanford University School of Medicine

**Keywords:** radiation exposure, physician ordering, computed tomography scanning

## Abstract

Objectives

To determine whether real-time passive notification of patient radiation exposure via a computerized physician order entry system would alter the number of computed tomography scans ordered by physicians in the Emergency Department (ED) setting.

Methods

When a practitioner ordered a computed tomography scan, a passive notification was immediately and prominently displayed via the computerized physician order entry system. The notification stated the following: the amount of estimated radiation in millisieverts (mSv), the equivalent number of single-view chest radiographs, and equivalent days of average environmental background radiation to which a patient during a specific computed tomography scan would be exposed. The primary outcome was changed in the number of computed tomography scans ordered when comparing data collected before and after the addition of the notification.

Results

Before the dosimetry notification (“intervention”) was instituted, 1,747 computed tomography scans were performed on patients during 11,709 Emergency Department visits (14.9% computed tomography scan rate). After the intervention had been instituted, 1,827 computed tomography scans were performed on patients during 11,582 Emergency Department patient visits (15.8% computed tomography scan rate). No statistically significant difference was found for all chief complaints combined (p = 0.17), or for any individual chief complaint, between the number of computed tomography scans performed on Emergency Department patients before versus after the intervention.

Conclusions

Passive real-time notification of patient radiation exposure displayed in a computerized physician order entry system at the time of computed tomography scan ordering in the Emergency Department did not significantly change the number of ordered scans.

## Introduction

Use of all types of diagnostic radiologic examinations in Emergency Departments (ED) in the United States has increased ten-fold between 1950 and 2006, with an estimated 67 million computed tomography (CT) scans completed in 2006, and a 330% increase in CT utilization from 1996 to 2007 [1-2]. In 2010, 80 million CT scans were performed, with a projected year-over-year increase of 10% [1]. In large part because of the increase in CT scan utilization, potential adverse effects of ionizing radiation originating in health care settings are a growing concern, especially in the pediatric population [3]. It is estimated that up to 2% of all cancers may be caused by radiation from medical CT scanning [4].

For many reasons, which include increasing patient visits, efficiency, inpatient physician and patient expectations, and litigation avoidance, ED physicians are under increasing pressure to accurately evaluate, diagnose, and treat increasing numbers of patients in an expeditious and thorough manner. Implementation of computerized physician order entry (CPOE) systems assists physicians in adapting to these pressures by decreasing costs, shortening the length of stay, increasing the timeliness of critical test result reports, and decreasing medical errors in the inpatient setting [5-6].

CPOE facilitates a study of physician ordering practices, use of clinical decision rules, and overall knowledge. However, there has not yet been a study to evaluate whether CPOE systems utilizing passive notification (defined as the display of information without requiring acknowledgment) can decrease unnecessary CT scans, and therefore radiation exposure, in patients in the ED setting.

The purpose of the present study was to determine whether real-time passive notification of patient radiation exposure via a CPOE system at the time of order entry would alter the number of CT scans requested by physicians in the ED setting. 

## Materials and methods

### Study design and setting

The study was performed at a tertiary academic medical center with level-one adult and pediatric trauma designations, a dedicated pediatric ED, and an annual census of more than 62,000 visits. The ED is staffed by board-certified adult and pediatric emergency physicians, emergency medicine residents, and residents from other specialties (e.g., surgery, internal medicine, obstetrics and gynecology, and pediatrics). The study was determined to be exempt from informed consent by the Stanford Institutional Review Board.

**Figure 1:**
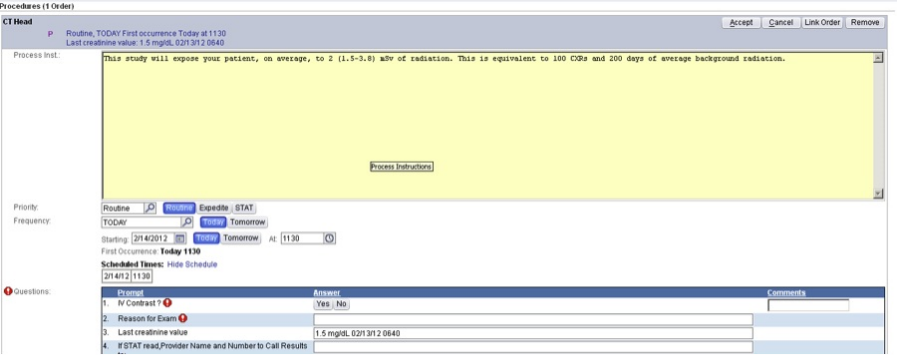
Example of Notification Bar as Displayed in EPIC The notification, in this case for a CT scan of the head, states, “This study will expose your patient, on average to 2 (1.5-3.8) mSv of radiation. This is equivalent to 100 chest X-rays and 200 days of average background radiation.”

The communication method to inform ordering practitioners of the estimated amount of radiation exposure for studies was to prominently post a notification via the existing CPOE system (EPIC, Verona, WI). This notification (the “intervention”), an example of which is shown in Figure 1, reported the amount of estimated radiation in millisieverts (mSv), equivalent number of single-view chest radiographs, and days of average environmental background radiation. The notification appeared immediately whenever a practitioner ordered a CT scan.

This study was powered to detect a 10% relative reduction in CT scan ordering or a 1.5% absolute reduction in CT scan ordering using a two-tailed significance level. This was based on a CT scan rate of 15 scans per 100 ED patients. To power and demonstrate this effect size, a sample size of 8,524 patients in each group was needed. A P-value of less than 0.05 was used to denote statistical significance. This number was chosen to be certain that the goal was met to have 80% power to detect a 10% relative drop in scan rates. The notification was added on May 17, 2012, to all login contexts within EPIC at the study hospital, including ED and inpatient settings. CT scan ordering data were then collected for 10,000 ED patient visits from March 10 to May 16, 2012. The increase in the number of subjects over the minimal number needed to power the study could be expected to increase power. Data was collected from the Stanford Translational Research Integrated Database Environment (STRIDE), which houses clinical information on patients seen at the study hospital since 1995.

### Study population

Participants in this study included all ED patients who underwent CT scanning during the study period of March 10 to July 23, 2012. They included adult, pediatric, trauma, and pregnant patients. Patients were excluded if they did not undergo CT scanning.

### Interventions and data collection and processing

To determine the estimated amount of radiation exposure for each study to be messaged to ordering physicians, radiation dosimetry data for each type of included CT scan were analyzed. These data were reviewed and analyzed by the study hospital’s Department of Radiology. The method was to review 14 days of CT scan dosimetry data for each type of study ordered for patients in the ED. If fewer than 20 CT scans of a particular type were performed in the 14 day period, the period was continued until 20 CTs scans were completed. The mean amount of radiation in millisieverts (mSv) was calculated for each CT scan type. Because the dose of radiation administered to each patient for the same diagnostic test is not the same, doses were calculated to within confidence intervals of 95%. These determinations (Table 1) served as the basis for the notifications placed in the CPOE system that informed ordering physicians of the amount of radiation to which each patient would be exposed by a particular type of CT scan.

**Table 1:** Radiation Dosimetry Data with Confidence Intervals for Each CT Type These data served as the basis for each EPIC notification.

Radiation Dosimetry Data with Confidence Intervals for Each CT Type			
CT Type	mSv (Mean with 95% CI)	CXR Equivalents	Equivalent Days of Average Background Radiation
CT Head	2 (1.5-3.8)	100	200
CT Head, Cervical Spine	4 (3.1-7.1)	200	400
CT Cervical Spine	3 (2.4-6.9)	150	300
CT Head, Facial Bones, Cervical Spine WO Contrast	7 (5.2-11.1)	350	700
CT Head Perfusion W Contrast	15 (9.7-21.0)	750	1500
CT Angio Head	5 (3.9-8.5)	250	500
CT Angio Head and Neck	9 (7.0-13.6)	450	900
CT Pulmonary Embolism	15 (9.1-22.4)	750	1500
CT Pulmonary Embolism and Lower Extremity	18 (13.6-27.6)	900	1800
CT Abdomen	8 (5.3-16.9)	400	800
CT Abdomen and Pelvis	12 (8.5-20.7)	600	1200
CT Angio Abdomen and Pelvis	15 (11.0-26.2)	750	1500
CT Angio Chest	25 (18.9-32.0)	600	1200
CT Angio Chest and Abdomen	12 (8.5-20.7)	1250	2500
CT Angio Chest Abdomen and Pelvis	26 (18.9-32.0)	1300	2600
CT Pelvis	6 (4.7-9.1)	300	600
CT Lumbar Spine	5 (4.0-8.3)	250	500
CT Thorax	7 (4.9-12.2)	350	700
CT Thoracic Spine	6 (4.7-11.9)	300	600

Data were collected from the hospital’s electronic medical record (EPIC). These data included radiologic tests ordered, estimated ionized radiation administered to patients, final patient disposition, and length of stay. Retrospective data were then extracted from the STRIDE database.

Patient nonclinical demographics were de-identified by STRIDE. Each patient was assigned a “de-identified patient number,” and each visit was assigned a “de-identified patient encounter number.” For each patient ED visit, the associated chief complaint, ICD-9 diagnosis code, admitting department, and CT scan ordered. For each CT scan, a “de-identified order number” was assigned and associated with a particular ED visit. Summary impressions, which are standardized codes that indicate the significance of the diagnostic findings contained within the study, were extracted for each CT scan.

Chief complaints from each visit during the study period were assigned to one of 20 chief complaint categories. Sixteen percent (3,224) of visits contained more than one chief complaint; these visits were assigned a single chief complaint based on the complaint most likely to have triggered the CT scan order. For 164 of these 3,224 (Table 2) visits with multiple complaints, the assignment was based upon the actual CT scan ordered because more than one of the chief complaints could potentially have triggered the order. Six hundred and twenty-four visits did not have a chief complaint recorded; these visits were excluded from the overall analysis.

**Table 2:** Chief Complaint Categories, Frequencies, and Percentages

Chief Complaint Categories, Frequencies, and Percentages				
Category	Frequency	Percent	Cumulative Frequency	Cumulative Percent
Abdominal Pain	2510	11.07	2510	11.07
Altered Mental Status	1425	6.29	3935	17.36
Cardiovascular	1645	7.26	5580	24.62
Edema	220	0.97	5800	25.59
Environmental	91	0.4	5891	25.99
Gastroenterology	1752	7.73	7643	33.72
Genitourinary	728	3.21	8371	36.93
Headache	692	3.05	9063	39.98
Hematologic	102	0.45	9165	40.43
Infectious	2427	10.71	11592	51.14
Malignancy	31	0.14	11623	51.28
Musculoskeletal Pain	2396	10.57	14019	61.85
Neurologic	952	4.2	14971	66.05
Obstetrics-Gynecology	365	1.61	15336	67.66
Other	1633	7.2	16969	74.86
Psychiatric	844	3.72	17813	78.59
Pulmonary	1176	5.19	18989	83.77
Skin	563	2.48	19552	86.26
Surgical Complication	282	1.24	19834	87.5
Trauma	2833	12.5	22667	100
Frequency Missing = 624 with no chief complaint recorded				

### Outcome measures

The primary outcome of this study changed in the number of CT scans ordered when comparing data collected before and after the addition of the radiation dosimetry notification delivered at the time of order entry via EPIC.

### Data analysis

To assess the impact of displaying dosimetry information, the rates of images ordered, both overall and as a function of chief complaint body system, before and after the intervention were assessed. For patients with multiple chief complaints, two authors (Lauren AP, Paul SA) reviewed the image orders and attributed each imaging study to a single complaint. Frequency counts of images done, both overall and by chief complaint and body system, before and after the intervention were calculated and the rates were compared using Poisson regression methods. A priori, the body system for which the study was ordered was deemed most likely to be impacted by the availability of dosimetry information and was therefore considered a primary endpoint. Other sites were considered as secondary. Therefore, these exploratory analyses were not subject to a correction for multiple comparisons. Tertiary analyses explored the overall rates for finding abnormalities. Rates of findings (categorized as non-diagnostic, no significant abnormality, previously reported abnormality, possibly significant abnormality, and critical result) before and after the intervention were assessed. Differences in these rates were evaluated using the Freeman-Halton extension of Fisher's Exact test. All analyses were done in SAS 9.4. Two-tailed p-values less than 0.05 were considered statistically significant.

## Results

No statistically significant difference was found for any chief complaint between the number of CT scans performed on ED patients, before and after the intervention introduced by this study (Tables 3, 4). Before the intervention was instituted, 1,747 CT scans were performed on patients during 11,709 ED visits. This represents a 14.9% CT scan rate. After the intervention had been instituted, 1,827 CT scans were performed on patients during 11,582 ED patient visits. This represents a 15.8% CT scan rate. No statistically significant difference was found between these two periods for all chief complaints during all visits (p = 0.17). Similarly, no statistically significant difference was found between the number of CT scans performed before versus after the intervention on patients in any of 20 chief complaint categories. P-values ranged from 0.08 to 0.93. For example, for patient visits in the chief complaint category of “Trauma,” 590 CT scans were performed during 1,552 patient visits (38.0% CT rate) before the intervention, and 741 CT scans were performed during 1,849 patient visits (40.1% CT rate; p = 0.93) after the intervention. For patient visits in the chief complaint category of “Abdominal Pain”, 104 CT scans were performed during 1,262 patient visits (8.2% CT rate) before the intervention, and 84 CT scans were performed during 1,248 patient visits (6.7% CT-rate; p = 0.29) after the intervention. For patient visits in the chief complaint category of “Headache,” 109 CT scans were performed during 383 patient visits (28.5% CT rate) before the intervention, and 105 CT scans were performed during 309 patient visits (34.0% CT rate; p = 0.08) after the intervention.

**Table 3:** Number and Percent of CT scans Ordered Before and After the Intervention by Type of CT Scan

CT Scan Frequency and Percentage by CT Scan Type				
Description		Time		
		Before	After	Total
CT ABDOMEN	Frequency	6	1	7
	Percent	85.71	14.29	
CT ABDOMEN AND PELVIS	Frequency	188	187	375
	Percent	50.13	49.87	
CT ANGIO ABDOMEN RUNOFF	Frequency	3	3	6
	Percent	50	50	
CT ANGIO CHEST	Frequency	50	85	135
	Percent	37.04	62.96	
CT ANGIO CHEST AND ABDOMEN	Frequency	0	2	2
	Percent	0	100	
CT ANGIO CHEST ABDOMEN AND PELVIS	Frequency	18	19	37
	Percent	48.65	51.35	
CT ANGIO HEAD	Frequency	6	3	9
	Percent	66.67	33.33	
CT ANGIO HEAD AND NECK	Frequency	44	53	97
	Percent	45.36	54.64	
CT HEAD	Frequency	899	884	1783
	Percent	50.42	49.58	
CT HEAD AND CERVICAL SPINE	Frequency	209	240	449
	Percent	46.55	53.45	
CT HEAD FACIAL BONES AND CERVICAL SPINE	Frequency	39	64	103
	Percent	37.86	62.14	
CT PELVIS	Frequency	33	36	69
	Percent	47.83	52.17	
CT PULMONARY EMBOLISM AND LOWER EXTREMITY	Frequency	10	6	16
	Percent	62.5	37.5	
CT PULMONARY EMBOLISM CHEST ONLY	Frequency	171	157	328
	Percent	52.13	47.87	
CT CERVICAL SPINE	Frequency	61	65	126
	Percent	48.41	51.59	
CT LUMBAR SPINE	Frequency	79	80	159
	Percent	49.69	50.31	
CT THORACIC SPINE	Frequency	69	70	139
	Percent	49.64	50.36	
CT THORAX	Frequency	95	85	180
	Percent	52.78	47.22	
TOTAL		1980	2040	4020

**Table 4:** Number of CT scans performed before and after the intervention by chief complaint category This table also shows the number of patients evaluated within each chief complaint category before and after the intervention. A p-value of <0.05 when comparing the rates of CT scans before and after the intervention was considered statistically significant.

Chief Complaint Category	Number of CTs Performed		Number of Patients with Chief Complaint		p value
	Before	After	Before	After	
Abdominal Pain	104	84	1262	1248	0.29
Altered Mental Status	178	183	721	704	0.3
Cardiovascular	131	131	838	823	0.93
Edema	3	3	100	120	0.11
Environmental	1	1	25	66	0.5
Gastrointestinal	466	54	881	872	0.33
Genitourinary	40	44	352	376	0.59
Headache	109	105	383	309	0.08
Hematologic	3	5	47	55	0.92
Infectious	47	41	1357	1075	0.76
Malignancy	1	3	16	16	0.31
Musculoskeletal Pain	81	89	1180	1216	0.61
Neurologic	221	185	511	454	0.4
Obstetrics-Gynecology	2	1	168	197	0.47
Other	36	30	854	797	0.68
Psychiatric	39	28	422	422	0.17
Pulmonary	106	97	642	536	0.57
Skin	1	2	261	302	0.65
Surgical Complication	8	3	137	145	0.1
Trauma	590	741	1552	1849	0.93
All Complaints	1747	1827	11709	11582	0.17

## Discussion

CT utilization in inpatient and outpatient health care settings, and especially in the ED setting, continues to increase. National CT use in EDs increased 330% from 1996 to 2007 [2, 4, 7]. At one United States tertiary care hospital from 2000 to 2004, CT use increased 27% in the outpatient setting and 48% in the inpatient setting, while ED CT uses increased 131% [7]. As a result of this increase in CT scanning, patients are receiving increasing doses of radiation from diagnostic and therapeutic medical imaging. The United States per capita annual effective radiation dose from medical procedures increased six-fold from 0.5 mSv in 1980 to 3.0 mSv in 2006 [1]. In our study, the average CT of the abdomen and pelvis exposed patients to 15 mSv of radiation, while the average CT angiogram imaging of the chest, abdomen, and pelvis delivered 26 mSv of radiation.

Multiple studies, based on radiation exposure rates of Japanese atomic bomb detonation survivors, have demonstrated a theoretical increased cancer risk attributable to exposure to even low-dose radiation. The Board on Radiation Effects Research VII report states that a single population dose of 10 mSv of radiation is associated with a lifetime attributable risk of 1 in 1000 of developing solid cancer or leukemia [8]. This dose of 10 mSv is well within the range of radiation doses delivered by modern CT scanners. Many patients have or will receive multiple CT scans during their lifetimes. For example, a patient with a history of pulmonary embolism (PE) who presents repetitively to EDs with symptoms suspicious for PE may undergo multiple chest radiographs and CT pulmonary angiogram studies. Each CT scan would expose the patient to on average 15 mSv of radiation, more than the amount that has been observed to increase the lifetime risk of cancer. Pediatric patients, who are ten times more sensitive to radiation than are adults, are undergoing more CT scans due to increased speed of CT scanning and decreased the need for sedation, among other factors [3, 9].

Two studies have demonstrated an increased risk of radiation exposure, which is actual and rather not theoretical. Both studies included children and adolescents. The first assessed the risk from CT scan exposure in persons from birth to age 22 years in the United Kingdom from 1985 to 2002, excluding patients previously diagnosed with a malignancy [10]. This study found an excess relative risk of 0.036 per mSv for leukemia and 0.023 per mSv for brain malignancy [10]. The second study evaluated the risk from CT scan exposure in patients from birth to age 19 years in Australia and found a 24% relative higher cancer incidence in CT scan-exposed patients [11]. Average effective radiation dose in this study was 4.5 mSv, and an absolute excess incidence for all cancers was 9.38 per 1,00,000 person years at risk [11]. These studies and logic suggest that as CT scan utilization increases, so will the overall population risk of developing cancer from radiation exposure.

Several studies demonstrated that physicians consider CT overutilization to be a problem and desire decision support to guide ordering practices [12 - 14]. Despite this desire and the fact that physicians are the persons ordering CTs, their knowledge of CT radiation doses and the associated cancer risk is not adequate [12, 15-16]. At the same time, physicians, especially emergency physicians, are under pressure to evaluate efficiently and accurately diagnose increasing numbers of patients. To improve efficiency, charting, and ease of ordering, CPOE systems embedded in electronic medical records (EMR) are instituted at many hospitals. CPOE systems have been shown to improve patient care and overall safety, being especially effective at improving provider adherence to guidelines [5-6]. CPOE systems also create the opportunity to embed standard-of-care guidelines to aid physicians in ordering and patient care. Despite this, few studies have evaluated the effects of adding guidelines for radiologic ordering to CPOEs. One recent study placed a passive reminder in its CPOE system to inform physicians if the patient had undergone five or more CT scans in the last 365 days; no significant change in the absolute number or rate of CT scan ordering was found [17].

In the present study, a passive notification was embedded into the order entry component of EPIC. This notification, at the time of a request for a CT scan, informed the attending and resident physicians of the quantity of radiation to which their patient would be exposed. We wished to evaluate if this specific message, method and timing of communication, and type of intrusion into the workflow process would influence ordering behavior. We did not observe a significant difference in the number of CT scans ordered from the ED related to the intervention.

The above study sufficiently powered to detect even a small difference in CT scan ordering between the pre- and post-intervention groups. We propose several reasons for not finding a statistically significant difference. First, the notification embedded into the CPOE system was passive rather than active. Attending and resident physicians were not required to acknowledge the notification before placing a CT scan order. Therefore, physicians may not have noticed or read the information, found it to be of little or no value at that particular moment (e.g., appeared too late) in their decision-making process, or decided that the benefits of ordering a CT scan outweighed the radiation exposure risks.

Our study was conducted at a level-one trauma center. Trauma patients overall undergo more diagnostic imaging, including more CT scans, and therefore are exposed to more radiation than are non-trauma patients [2]. In the current study, the CT scan rate for all patients with non-trauma-related chief complaints was 11.4% before the intervention and 11.1% after the intervention, while for trauma patients, it was 38% before the intervention and 40.1% after the intervention. In our hospital, we observe little or, more commonly, no discussion of radiation exposure during patient trauma treatment.

The current study showed that passively written notation about radiation exposure displayed to an ordering ED physician in the manner that we deployed did not decrease CT scan utilization. If we order too many tests, and some of these tests may reasonably be foregone, and if knowledge of radiation exposure might sway decision making, then we need to find a more effective way to interact with the ordering physicians. Algorithms designed to expedite workflow processes that include CT scans perhaps lead busy doctors attempting to manage a hectic ED away from contemplating the risk: benefit ratio of any individual test.

It is possible that mandatory acknowledgment of radiation exposure before placing an order will affect behavior. Behavioral interventions that include accountable justification and peer comparison have been shown to lower inappropriate antibiotic prescribing in the primary care setting [18]. As shown in this study, passive notification alone does not influence ordering behavior, active types of interventions deserve to be studied for CT scan ordering in the ED setting.

Several studies have demonstrated poor physician knowledge of radiation dosages from CT scans and the risks associated with this radiation. These studies also determined that physicians view CT scan overutilization as a problem and welcome guidelines to reduce CT scan ordering [12-13, 15-16]. It is our observation, along with others, that ED physicians generally believe that advanced imaging is overutilized [19]. It is our empirical observation that they further believe that CT scans lead to unnecessary radiation exposure and increased health care costs. However, changing ordering behavior has not been solved.

If we wish to influence physicians to order fewer CT scans, for reasons of patient safety or otherwise, then we need to find a solution. Perhaps there is a more persuasive messaging technique that would cause them to order safer. The messaging could include, e.g., less radiation exposure, substitutes, e.g., ultrasound, magnetic resonance imaging, clinical observation, provide more precise and persuasive indications for CT scan ordering, e.g., promulgation of clinical decision support rules, and in a useful fashion. Such messaging would give practicing ED clinicians a fighting chance of integrating all of this knowledge into their standard workflow processes.

### Limitations

Certain limitations may have influenced this study. It is possible that education and advertising needed to be conducted in advance of the intervention for it to become effective. It is possible that a more prominently displayed notification in the EMR might have been more effective. It is possible that the physicians might not understand the significance of radiation exposure as it was presented.

We were not able to control for the fact that while attending (faculty) physicians remain constant, different groups of residents rotate through the ED each month, including EM and off-service residents. It is possible that, had the entire physician group remained constant. This intervention might be effective after a sufficiently long enough period, that the providers became aware of and responsive to the information provided to them. It is possible that the two months pre- and post-intervention was too short a period to draw definitive conclusions. It may be that this intervention would require a longer period to become effective.

Patient volume and case mix in the ED fluctuates by time of year. Because the data collected in the pre- and post-intervention periods were not matched by time of year, it is possible that the type and number of patients in the ED at any given time may have influenced ordering behavior. This data matching is particularly important when considering the age of the patients as it relates to the propensity to order a CT scan.

Specific chief complaints were assigned to one of 20 chief complaint categories. Visits with multiple chief complaints were assigned to a single chief complaint category based on the complaint most likely to have triggered ordering of a CT scan.  Our determination of these assignments may have influenced the number of CTs attributed to each chief complaint category. It is also possible that the assignments were incorrect, although we do not believe this to be the case.

It is possible that the intervention occurred too late in the decision-making process, perhaps after other clinical decision rules had been deployed to determine the need for a CT scan. We did not control for whether or not a clinical decision rule was used. Perhaps radiation exposure will not be effectively addressed unless it is a factor integrated into a clinical decision rule.

We did not control for the precise time of availability of other imaging modalities, such as MRI or ultrasound, but during the period of this study, modalities which acted as alternatives to CT scan were readily available.

This study was performed at an urban academic ED. It may not be generalizable to the community or rural hospitals, or to other inpatient or outpatient settings. As noted above, factors that might improve the ability of a “passive” notification system to be helpful in decreasing the number of CT scans ordered are: a stable staff, a different visual design (e.g., red flashing warning), mandatory acknowledgment or acceptance of the notification (accountable justification) before being allowed to proceed with placing a test order, periodic feedback to individual ordering physicians of their ordering profiles i.e., either in isolation or in comparison to peers, a different set of guiding information (perhaps to include the financial expense of the studies), or presentation of clinical pathways or decision rules (e.g., risks versus benefits). 

## Conclusions

Real-time passive notification of patient radiation exposure displayed in a CPOE system at the time of CT scan ordering in the ED did not significantly change the number of ordered scans. It remains to be determined whether or not there is a notification or other method that would cause physicians to order fewer CT scans in the ED setting.
